# Cost‐effectiveness of atraumatic restorative treatment combined with the Hall Technique for managing dental caries in remote Indigenous children

**DOI:** 10.1111/adj.13066

**Published:** 2025-03-14

**Authors:** U Tonmukayakul, S Kularatna, S Piggott, D Atkinson, D Brennan, L Jamieson, P Arrow

**Affiliations:** ^1^ Deakin Health Economics, Institute for Health Transformations Deakin University Geelong Victoria Australia; ^2^ Australian Centre for Health Services Innovation, School of Public Health and Social Work Queensland University of Technology Brisbane Queensland Australia; ^3^ Western Australia Dental Health Services Perth Western Australia Australia; ^4^ Rural Clinical School of Western Australia University of Western Australia Broome Western Australia Australia; ^5^ Australian Research Centre for Population Oral Health, Adelaide Dental School The University of Adelaide Adelaide South Australia Australia; ^6^ Dental School University of Western Australia Perth Western Australia Australia

**Keywords:** Indigenous oral health, atraumatic restorative treatment, Hall Technique, cost‐effectiveness, oral health disparities, dental caries management, culturally appropriate care

## Abstract

**Background:**

Indigenous children in remote Australia face significant disparities in oral health and have limited access to dental care. This study evaluated the cost‐effectiveness of Atraumatic Restorative Treatment combined with the Hall Technique (ART‐HT) compared to usual care.

**Methods:**

A cost‐effectiveness analysis using data from a 1‐year ART‐HT trial estimated dental caries status and costs from a health‐provider perspective. Two scenarios were examined: (1) actual treatment costs and (2) minimum dental services. The incremental cost per decayed, missing, and filled teeth (dmft) prevented was calculated in AUD 2021 prices. A probabilistic sensitivity analysis was conducted.

**Results:**

Among 228 children (ART‐HT = 122; usual care = 106), deterministic analysis showed ART‐HT required additional costs of $59.54 and $72.37 for scenarios 1 and 2, with 0.90 dmft prevented. Probabilistic sensitivity analysis revealed ART‐HT resulted in better oral health outcomes with a mean dmft prevented of 0.58 (95% uncertainty interval: 0.09 to1.07). The mean additional cost per dmft prevented was $118.50 and $181.84 for scenarios 1 and 2.

**Conclusions:**

ART‐HT effectively managed dental caries in Indigenous children, providing better oral health outcomes compared to usual care, albeit with modest additional costs. This approach may improve access to culturally appropriate dental care in remote communities.

**Abbreviations and acronyms**
ART‐HT: Atraumatic Restorative Treatment combined with the Hall Technique
DVA: Department of Veteran Affairs
GEE: generalized estimating equation
GIC: glass ionomer cement
ICER: incremental cost‐effectiveness ratio
PPHs: potentially preventable hospitalisations
WA: Western Australia

**Clinical Relevance**

Providing accessible and culturally appropriate dental care for Indigenous children in remote areas is a challenge. This study demonstrated the clinical and economic advantages of Atraumatic Restorative Treatment combined with the Hall Technique (ART‐HT) in managing dental caries among Indigenous children in under‐resourced settings. The ART‐HT approach is non‐invasive, culturally respectful, and can be seamlessly integrated into community‐based dental practices without the need for advanced dental facilities. This model could significantly improve oral health outcomes, while also addressing logistical challenges and inequalities in accessing usual dental care in remote communities.

## INTRODUCTION

Young children and adolescents living in rural and remote geographic locations in Australia have poorer oral health than those living in metropolitan areas.^1^ This is thought to be due in part to poor access to care in both rural and remote locations. In Western Australia (WA), Aboriginal and Torres Strait Islander children (hereafter referred to as Aboriginal) had six times more decayed tooth surfaces than non‐Aboriginal children, with approximately two‐thirds of their decayed teeth being untreated.^2^ In addition, the rate of potentially preventable hospitalisations (PPHs) associated with dental conditions in WA was almost double among Aboriginal children (4.8 vs. 2.9 per 100 000) in 2017–2018.^1^ PPHs, defined as conditions that would not result in hospitalization if adequate and timely non‐hospital care were received, often include dental treatments under general anaesthesia.^1^ The cost of hospitalization for dental treatments in WA has been estimated at $AUD9 million per annum.^3^ Minimizing the rate of PPHs is one of the key goals under the National Healthcare Agreement between the Australian Government and the State and Territory Governments^4^ and the Australian National Oral Health Plan 2015–2024.^5^

The conventional approach to dental caries management in young children relies on the use of local anaesthesia and dental drills in clinical settings. Occasionally, the condition is managed under hospital‐based general anaesthesia. In Aboriginal communities, access to the usual care model is limited because it depends on *ad hoc* visits to communities by dental providers or travel to community‐based dental clinics in distant locations. Inadequate resources and limited cultural awareness by service providers also contribute to the limitations in service delivery.^3^, ^6^, ^7^

The minimally invasive atraumatic restorative treatment and Hall technique (ART‐HT) is an alternative service model that can overcome access to care. ART has a long history of use. It was established in 1986 in response to a lack of dental services and poor access to electricity and water in outreach locations.^8^, ^9^ ART is underpinned by the minimal intervention dentistry concept, where hand instruments are used to remove soft carious lesions, and the prepared cavity is restored with glass ionomer cement (GIC). ART also includes oral education, prevention, and promotion components such as oral hygiene, diet instructions, and pit and fissure sealants.^10^ Evidence shows the benefits of ART in reducing patient discomfort, pain, and anxiety.^11^, ^12^ The survival of ART fillings in primary teeth in field settings is as high as that in dental clinics.^13^ HT focuses on the restoration technique of removing gross debris using hand instruments, followed by placing prefabricated metal crowns with glass ionomer cement.^14^, ^15^ HT restorations have incidence rates of pain and/or infections similar to those of conventional restorations.^16^

ART‐HT delivered in a primary care setting has been tested in metropolitan/urban settings in WA and has been shown to reduce the referral rate for specialist care, improve quality of life, reduce dental anxiety, and save costs for dental service providers.^17^, ^18^, ^19^, ^20^ However, the value of the application of ART‐HT in remote communities was unknown prior to the current research. This study presents a cost‐effectiveness analysis using data collected from an ART‐HT cluster‐randomized trial in remote Aboriginal communities in WA. Findings with respect to the impacts of the ART‐HT trial on clinical measures, oral health‐related quality of life, and child anxiety have already been reported.^17^, ^18^

## MATERIALS AND METHODS

### ART‐HT trial

The ART‐HT trial was a parallel, stepped‐wedge, assessor‐blinded, cluster‐randomized trial conducted in remote and rural Aboriginal communities in the Kimberley region of WA. An Aboriginal reference group with members drawn from health organizations in the Kimberley was formed to guide the design and implementation of the study.

Consenting communities were randomly allocated to the ART‐HT or usual care groups. Full details of the study design, inclusion–exclusion criteria, and adaptations, the withdrawal of one community, randomisation strategy, group allocation blindness, exclusion of the Child Health Utility 9 dimensions at the follow‐ups, changing the follow‐ups from 6 to 12 months, and analyses of clinical and economic outcomes have been reported elsewhere.^18^, ^21^

This trial was registered in the ANZ Clinical Trials Registry (ACTRN126001537448). Ethics approval was granted by the WA Country Health Service Human Research Ethics Committee (WACHS HREC Project References 2017/01), WA Aboriginal Health Ethics Committee (Project Reference: 790), University of Adelaide Health Research Ethics Committee (H‐2017‐015), and the Deakin Health Human Research Ethics Committee (2022‐084). Parents or carers of all participants provided signed informed consent.

### Participants

Eligible participants were Aboriginal children younger than 6 years living in participating communities. Children with medical or developmental conditions that limited dental treatment in a primary care setting or acute dental infections that required urgent dental care were excluded.

### Intervention and control

The ART‐HT procedures were performed by school dental therapists who had completed the ART‐HT training. Fluoride varnish applications, along with other preventive measures, were implemented to slow the progression of caries. The cavitated lesions were cleaned using hand instruments and restored with glass‐ionomer cement. For HT crowns, minimal tooth preparation was performed before seating and luting the prefabricated crowns with glass‐ionomer material. When necessary, the decision to use a dental drill was based on the child's clinical needs and their ability to tolerate the procedure. A portable X‐ray machine was used to obtain pre‐treatment radiographs before HT treatment and in cases where pulp therapy and dental extraction were needed. These procedures were performed using portable equipment in various community settings, such as traditional local dental clinic settings, school libraries, or family day care centres. Parents and children also received guidance on brushing their teeth with fluoride toothpaste and toothbrushes.

Control participants were informed of their oral health status and advised to seek care from their usual sources. The usual care included visiting government dental services (expected visits three times per year), volunteer‐based non‐government dental services (intended to be annually), or Royal Flying Doctor Dental Services (intended to be at least annually), which provide services at no out‐of‐pocket cost. However, actual access to these services was often limited and inconsistent. While these providers typically use conventional dental restorative procedures involving the use of local anaesthesia and dental drills, such treatment was only available when children actively sought care and services were accessible – which often required travelling hundreds of kilometres to larger towns to seek either public or private dental services. The main outcome paper reported that while the control group participants had access to these providers between the baseline and follow‐up,^18^ actual utilization of services was minimal as evidenced by the low rate of restorative treatments received as 0.1 treatment per child.

The original research design was to offer a delayed ART‐HT intervention to control participants at follow‐up after an oral health assessment. However, hard Covid‐19 restrictions prevented the team from evaluating the effects of delayed ART‐HT and led to the trial's conclusion after 1 year.

### Outcome measures

This economic evaluation focused on assessing changes in decayed, missing, and filled primary teeth (dt, mt, ft) and combined teeth (dmft) from baseline to follow‐up. Inter‐examiner reliability for dental examinations was assessed using Cohen's Kappa statistic.

While the disaggregated changes in each caries index component provide richer information, the incremental cost‐effectiveness ratio (ICER) per dmft provides more robust information. The ICER is the ratio of the differences in costs and effects between ART‐HT and usual care. The ICER informs how much extra cost would be required (or saved) to gain an additional unit of health outcomes.

### Costs

Two cost components borne by healthcare providers were considered in the ART‐HT group: ART‐HT training ($9.24 per child) and treatment costs. The training cost per child was derived from the total training expenditure of $1635 (comprising venue rent $500, training material and preparation $158.10, trainer's time cost $324.30, and trainees' time cost $652.53), divided by the total number of participating children at baseline. These costs were obtained from the study records and financial documentation.

Although the delivery of the ART‐HT intervention involved travel costs and per diem allowances, they were excluded from the analysis due to the absence of publicly available cost data, particularly for usual care providers. Given the multiple providers involved in usual care delivery, each with different travel arrangements and cost structures, including partial travel cost data could potentially introduce bias into the analysis. Therefore, our analysis focused on direct intervention costs that could be reliably measured and compared between groups. Research‐related costs were excluded to reflect the real‐world implementation of services.

Two treatment cost scenarios were considered: (1) the actual treatment costs provided by test clinicians and (2) the costs of minimum preventive services that a clinician would provide to a child living in hard‐to‐reach communities with high dental caries prevalence. Cost scenario 2 assumed that all children with ART‐HT received oral health education and fluoride application.

Treatment costs were estimated from codes of dental procedures recorded in participant clinical notes based on the 2021 Department of Veteran Affairs (DVA) fee schedule to generalize the findings to other Australian States and Territories. The DVA fee schedule for dental services for children was used in preference to an alternative schedule under the Child Dental Benefit Scheme because the latter did not contain all the itemized codes collected from the trial.

Collecting detailed cost information about dental services provided by usual care providers presented significant challenges. The extreme geographical isolation of participating communities (some located hundreds of kilometres from major centres) and frequent adverse weather conditions made physical access impractical. Additionally, requesting detailed treatment cost information would have created an undue burden on participating children and families in these vulnerable communities. Therefore, this study used the average treatment cost per child provided by the WA Dental Health Services in the Kimberley region in 2018–19 and 2019–20 as a proxy. This source was selected because it represents actual service delivery costs in the same geographical region for the same population demographic. All costs, including these proxy costs, were adjusted to the Australian dollar 2021 price using standard inflation rates to ensure comparability.

### Cost‐effectiveness analysis

**Fig. 1:**
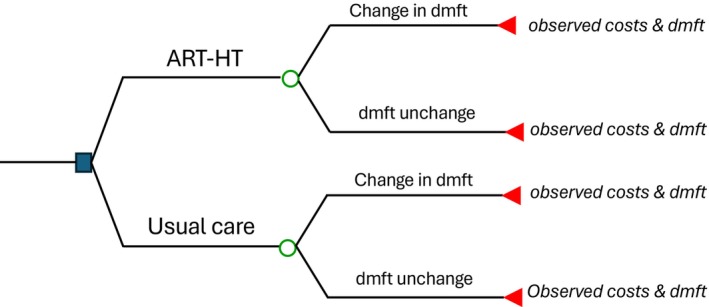
Decision analytic model.

A cost‐effectiveness analysis simultaneously compared the costs and effects of at least two alternatives using a simple decision tree (Fig. 1). The mean pre‐ and post‐caries indices of each group were compared to minimize the population confounders between the groups. In the base case or deterministic analysis, the incremental effects and costs between the ART‐HT and the control were estimated using the observed means.

In probabilistic sensitivity analysis, the observed means were replaced by values randomly selected from the mean and SE based on the distribution of each variable to calculate an incremental cost‐effectiveness ratio (ICER) and recorded as one iteration. These steps were repeated 1000 times using a bootstrapping approach to generate 1000 possible ICER iterations. Mean, range, and 95% uncertainty limits of the ICERs were determined. For treatment costs of the control group and ART‐HT training that do not have variations, their mean values were used as SE ranges in the probabilistic sensitivity analysis. The 1000 ICERs were plotted on a cost‐effectiveness plane where the *x*‐axis represents the difference in oral health improvement and the y‐axis represents the difference in costs between the ART‐HT and usual care.

The costs and effects were not discounted because the study timeframe was less than 1 year. Longer‐term projection of costs and effects was not attempted due to data limitations, which would have led to inaccurate estimates. Microsoft Excel was used to construct and analyse the cost‐effectiveness model.^22^ The model parameters are reported in Table 1.

**Table 1:** Model input parameters

Parameters	Mean (SE)	Distribution (Assumption)
Actual ART‐HT treatment costs	$470.90 (45.42)	Gamma distribution
ART‐HT treatment cost for hypothetical scenario	$483.73 (44.46)	Gamma distribution
Training cost per child	$9.24 (9.24)	Gamma distribution (SE = mean)
Average treatment costs under the usual care	$420.60 (40.85)	Gamma distribution
dmft change of the control group	0.4 (0.22)	Normal distribution
dmft change of the ART‐HT group	1.3 (0.13)	Normal distribution

## RESULTS

**Fig. 2:**
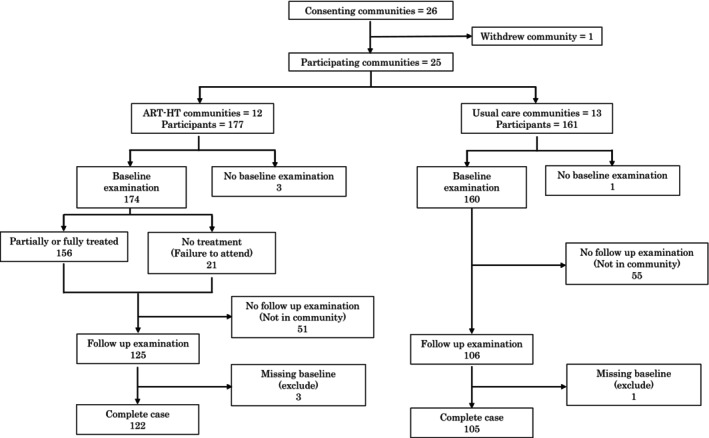
Participant flowchart.

There were 122 completed cases out of 177 children in the ART‐HT group and 105 out of 160 children in the control group (Fig. 2). The loss to follow‐up was mainly due to the participants being out of the community at the time of the assessment.

The inter‐examiner reliability assessment showed good agreement between examiners, with Cohen's Kappa values of 0.81 on dmft. Data from participants assessed at both baseline and follow‐up showed that initially, the ART‐HT group had an average of 4.4 dt and 0.2 ft, indicating a higher caries experience than the control group, which had 3.1 dt and 0.07 ft. Over the study period, the ART‐HT group showed a decrease in dt by an average of 0.6 teeth, decreasing from 4.4 to 3.8, and a significant increase in ft, from 0.2 to 1.2. In contrast, the control group had an increase in dt, rising from 3.1 to 4.1, with a slight increase in filled teeth, from 0.07 to 0.2. These changes suggested that the ART‐HT effectively reduced decay and increased restorative care compared to the usual care group.

### Economic evaluation results

Table 2 shows the cost‐effectiveness of the findings. ART‐HT delivery incurs additional costs. Deterministic analysis found that the ART‐HT intervention had higher total costs compared to usual care, with a difference of $59.54 in cost scenario 1 (observed treatment) and $72.37 in cost scenario 2 (minimum ART‐HT service).

**Table 2:** Deterministic and probabilistic sensitivity analysis cost‐effectiveness analysis results

Deterministic	Cost scenario 1 Observed treatment			Cost scenario 2 Minimum ART‐HT service		
	ART‐HT	Usual care	Differences	ART‐HT	Usual care	Differences
Costs						
Treatment costs	$ 470.90	$ 420.60		$ 483.73	$ 420.60	
ART‐HT training costs	$ 9.24	Nil		$ 9.24	Nil	
Total costs	$ 480.14	$ 420.60	$ 59.54	$ 492.96	$ 420.60	$ 72.37
Changes in dental caries indices between baseline and follow‐up within group						
*dt*	−0.73	0.99	−1.72			
*mt*	0.10	0.17	−0.07			
*ft*	1.03	0.11	0.92			
*dmft*	0.40	1.30	−0.90			
Incremental cost effectiveness ratio ($ per dmft prevented)	$66.14			$80.39		

Probabilistic	Cost scenario 1 Observed treatment			Cost scenario 2 Minimum ART‐HT service		
	ART‐HT	Usual care	Differences	ART‐HT	Usual care	Differences
Mean costs (95% uncertainty interval)						
Treatment costs	$ 471.00 ($383.60 to $563.20)	$ 420.45 ($418.66 to $422.22)		$ 482.29 ($390.27 to $579.71)	$ 420.45 ($418.66 to $422.22)	
ART‐HT training costs	$ 9.35 ($0.23 to $34.38)	Nil		$ 9.35 ($0.23 to 34.38)	Nil	
Total costs	$ 480.35 ($395.13 to $570.47)	$ 420.45 ($418.66 to $422.22)	$ 59.91 (−$25.09 to $150.70)	$ 491.64 ($395.85 to $590.54)	$ 420.45 ($418.66 to $422.22)	$ 71.19 (−$24.66 to $169.55)
Mean changes in dental caries indices between baseline and follow‐up (95% Uncertainty Interval)						
*dt*	−0.51 (−0.90 to −0.11)	0.65 (0.19 to 1.08)	−1.16 (−1.72 to −0.59)			
*mt*	0.07 (0.01 to 0.15)	0.11 (−0.01 to 0.29)	−0.04 (−0.26 to 0.11)			
*ft*	0.72 (0.50 to 0.94)	0.07 (0.00 to 0.16)	0.64 (0.42 to 0.87)			
*dmft*	0.28 (−0.03 to 0.58)	0.86 (0.45 to 1.24)	0.58 (1.07 to −0.09)			
Incremental cost effectiveness ratio ($ per dmft prevented)	$118.50 (−$69.24 to $558.82)			$181.84 (−$60.03 to $520.05)		

The negative dmft differences were inverted to positive values for ICER calculation to align with the conventional interpretation of the cost‐effectiveness results, where the larger positive value represents a greater improvement in oral health outcome.

Negative dt, mt, and dmft differences in Table 2 are desirable, as they represent improvements in dental caries status. Changes in dental caries indices between baseline and follow‐up showed notable improvements in the ART‐HT group. The ART‐HT intervention resulted in a decrease of 0.73 in dt, compared to an increase of 0.99 in the usual care group, leading to the dt prevented of 1.72. Children in the ART‐HT group received almost 10 times more restorative treatments than usual care (0.07 vs. 0.72 treatments per child). The overall dmft index had a smaller increase of 0.40 for ART‐HT compared to 1.30 for usual care, resulting in a favourable difference of 0.90 dmft prevented. These findings demonstrate prominent positive impacts of ART‐HT on dt, ft, and overall caries experience (dmft).

The deterministic ICERs indicated an additional cost of $66.14 per dmft prevented for scenario 1 and $80.39 for scenario 2. The probabilistic sensitivity analysis supported these findings, showing a mean total cost difference of $59.91 (95% uncertainty interval [UI]: −$25.09 to $150.70) for scenario 1 and $71.19 (95% UI: −$24.66 to $169.55) for scenario 2. The mean dmft difference was 0.58 (95% UI: 0.09 to 1.07) in favour of ART‐HT. The probabilistic ICER showed a mean additional cost of $118.50 (95% UI: −$69.24 to $558.82) per dmft prevented for scenario 1 and $181.84 50 (95% UI: −$60.03 to $520.05) for scenario 2.

**Fig. 3:**
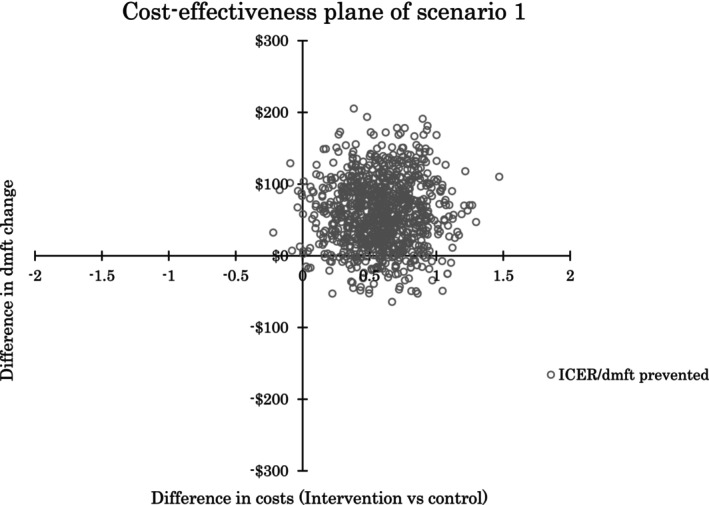
Cost‐effectiveness plane of scenario 1.

**Fig. 4:**
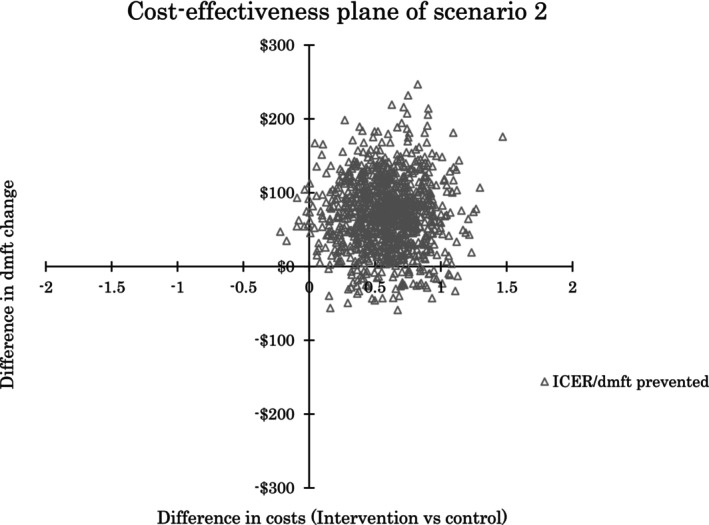
Cost‐effectiveness plane of scenario 2.

Figs 3 and 4 display 1000 ICER per dmft prevented plots for cost scenarios 1 and 2, respectively. The larger positive values on the x‐axis represent a greater improvement in oral health. It is important to note that while the ART‐HT intervention showed improved oral health outcomes, it came at an additional cost in most scenarios. The widespread of ICERs on the cost‐effectiveness planes suggests variability in the cost‐effectiveness results, ranging from potential cost‐savings to additional costs for the improved outcomes.

## DISCUSSION

This study presented the results of cost‐effectiveness modelling of the ART‐HT trial undertaken among preschool‐age children in remote Aboriginal communities in the Kimberley region of WA. Both deterministic and sensitivity analyses were conducted across two cost scenarios. The ART‐HT intervention demonstrated superior outcomes in managing dental decay with modest additional costs in most scenarios.

Deterministic analysis showed that the ART‐HT required an additional $66.14 and $80.39 to prevent one dmft for cost scenarios 1 and 2, respectively. Results from probabilistic sensitivity analysis revealed even more nuanced results, showing that the ART‐HT had a mean additional costs of $118.50 (95% UI: −$69.24 to $558.82) per dmft prevented in scenario 1 and $181.84 (95% UI: −$60.03 to $520.05) per dmft prevented in scenario2.

The findings from this study were consistent with previous research demonstrating that ART alone or combined with ART‐HT saved costs and was a cost‐effective intervention in the management of early childhood caries in non‐Aboriginal children.^18^, ^19^, ^20^, ^21^, ^23^ In our model, the average treatment costs in cost scenarios 1 and 2 were $470.90 and $483.73 per child, respectively, which were favourable compared with the costs incurred by the Dental Health Services WA in the provision of dental services for children in the Kimberley region in the financial years 2018–19 and 2019–20 ($561.65 and $546.61 per child, respectively).

There is no yardstick to justify how much per dmft prevented is considered cost‐effective. The treatment cost per tooth may be used as a reference; however, treatments for carious lesions vary, making it challenging to identify the appropriate reference price. This leaves the judgment of “worthy” to the decision‐maker. From a cultural appropriateness and equity viewpoint, ART‐HT should be endorsed because it is likely to be applicable to other remote Aboriginal communities where direct health care costs are of interest.

In this study, the ART‐HT intervention demonstrated improved value for money, increasing levels of care (more prevented caries, and more restored teeth, and fewer extractions) compared to usual care, where access to consistent and timely dental treatment is often limited for children in remote Aboriginal communities. Although the quantum of change is relatively small, changes in these indices in participants who have limited access to dental care (the usual care group) would likely become more favourable, particularly if ART‐HT is implemented as an ongoing program.

According to the 2018–19 National Aboriginal and Torres Strait Islander Health Survey, 19% of Indigenous children aged 2 years or older did not go to a dentist at established clinics when they needed to in the past 12 months. Prohibiting reasons include costs, long wait times, or an appointment not being available at the time required.^24^ While community‐based dental services could solve the limited access to dental care issues in remote communities in WA's Kimberley region (study sites), the physical presence of oral health professionals is still required to deliver services in the community.^25^ Studies have demonstrated that the integration of dental and primary healthcare increases access to oral healthcare and positively influences oral health outcomes in young Australian and New Zealand children.^26^ This supports the use of alternative modes of service delivery, such as teledentistry and/or non‐invasive dental caries management (such as the application of silver fluoride), performed by trained non‐dental health professionals who live locally.

Our analysis demonstrates that the culturally appropriate interventions can achieve both clinical effectiveness and economic efficiency. The ART‐HT intervention specifically addressed geographical and cultural challenges faced by rural Aboriginal communities through community‐based delivery using portable equipment, eliminating travel barriers. Focus group interviews revealed that child‐centred care delivered in culturally safe settings was central to the intervention's acceptance.^25^ The high community participation rate was achieved through meaningful engagement with the Aboriginal Reference Group and extensive community outreach. These implementation features not only enhanced cost‐effectiveness but also ensured cultural appropriateness and sustainability. Our findings provide valuable insights for policy makers in other countries with remote Indigenous populations, such as Canada, Brazil, and New Zealand, demonstrating how economic efficiency can be achieved while respecting cultural needs and community preferences.

The ART‐HT trial faced several limitations, primarily due to the COVID‐19 lockdowns in which external visitors were strictly prohibited. Consequently, the study was allowed only one follow‐up visit and was ceased after 1 year. Approximately one‐third of the participants were lost to follow‐up as they were not present in their communities during the assessment visits. Although early community engagement was part of the trial, it did not mitigate the high mobility inherent in many Aboriginal community cultures. Despite this loss to follow‐up, Arrow *et al*. found that the characteristics of participants who were followed up were similar to those who were lost and comparable between the two groups, suggesting minimal bias in the results.^18^ There were some differences in baseline characteristics between the ART‐HT and usual care groups. However, the collected data were sufficient for evaluating the effectiveness of the intervention. Clinically meaningful changes in dt and ft were statistically significant in the ART‐HT group after adjusting for the baseline differences. Robust analytical methods, including multiple imputations of missing data and generalized estimating equation (GEE) to account for the clustering effect, were employed in the analysis of the primary outcome.^18^ GEE analysis, which controlled for baseline dental caries experience, age, sex, and water fluoride levels in the participating community, showed a 40% higher likelihood of dt (RR = 1.4, *P* = 0.02) and an 80% lower likelihood of having ft (RR = 0.20, p < 0.001) at follow‐up for children in usual care.^18^

The economic evaluation was limited to a 1‐year time horizon due to the paucity of evidence on caries progression and treatment longevity specifically in Aboriginal children. While the longer‐term projections could potentially provide valuable information for policy makers, extrapolating beyond the trial period without robust supporting evidence about long‐term costs and effectiveness could lead to unreliable conclusions. Future research should focus on gathering longer‐term data to enable more extended projections.

Another limitation is around the choice of modelling approach. While microsimulation modelling could have offered additional advantages in controlling for confounders between groups, the bootstrapping technique was chosen for several key reasons. First, the trial had a relatively short time horizon (less than 1 year) and focused on direct intervention effects, making the additional computational complexity of microsimulation less justified. Second, our bootstrapping approach was sufficient for capturing the key uncertainties in our analysis while maintaining model parsimony. Microsimulation models require substantial time and resources to develop and maintain, and given our study's scope and available data, the additional complexity may not have provided proportional benefits. The current analysis demonstrated a high probability of effectiveness, and the bootstrapping approach adequately addressed the key uncertainties in our study context.

In conclusion, this study assessed the cost‐effectiveness of a culturally appropriate ART‐HT compared with usual care in managing early childhood caries in Indigenous Australians living in remote communities from the health‐provider perspective. The ART‐HT intervention demonstrated better oral health outcomes with a mean dmft improvement of 0.58 (95%UI: 0.09 to 1.07), requiring modest additional initial costs of $59–72 per child. While not cost‐saving in all scenarios, the intervention showed potential cost‐effectiveness with ICERs of $118.50 and $181.84 per dmft prevented for actual treatment and minimum ART‐HT service scenarios, respectively. These findings suggest that the ART‐HT is a viable option for addressing dental care needs in remote communities, particularly in locations with limited access to conventional dental services. The improved oral health outcomes and cultural appropriateness may justify the additional investment, especially when considering the unique challenges faced by Aboriginal children in remote areas. Further research into long‐term cost‐effectiveness and potential modifications to reduce costs could enhance understanding of this promising intervention's sustained impact.

## AUTHOR CONTRIBUTIONS

**U Tonmukayakul:** Conceptualization; methodology; writing – original draft; writing – review and editing; visualization; formal analysis; validation. **P Arrow:** Conceptualization; funding acquisition; data curation; project administration; visualization; writing – review and editing; validation. **L Jamieson:** Investigation; writing – review and editing; validation. **S Piggott:** Funding acquisition; data curation; project administration; resources; writing – review and editing. **S Kularatna:** Investigation; writing – review and editing; visualization; validation. **D Brennan:** Investigation; writing – review and editing; validation. **D Atkinson:** Investigation; validation; writing – review and editing.

## FUNDING INFORMATION

The study was funded by the Australian National Health and Medical Research Council (APP11211982), Colgate Oral Care Australia, and the support of the Western Australia Dental Health Services.

## CONFLICT OF INTEREST

None of the authors have any conflicts of interest to declare.
